# Emerging Role of Essential Oils as Modulators of the JAK/STAT Signaling Pathway: A Comprehensive Review

**DOI:** 10.3390/ph19071006

**Published:** 2026-06-29

**Authors:** Maria Rosaria Perri, Carmine Lupia, Mary Fucile, Claudia-Crina Toma, Mariangela Marrelli, Giancarlo Statti, Filomena Conforti

**Affiliations:** 1 Department of Pharmacy, Health and Nutritional Sciences, University of Calabria, 87036 Rende, Italy; mary.fucile@unical.it (M.F.); mariangela.marrelli@unical.it (M.M.); filomena.conforti@unical.it (F.C.); 2 Mediterranean Ethnobotanical Conservatory, 88054 Sersale, Italy; studiolupiacarmine@libero.it; 3 National Ethnobotanical Conservatory, 85040 Castelluccio Superiore, Italy; 4 Pharmacognosy Department, Faculty of Pharmacy, Vasile Goldis Western University of Arad, 310045 Arad, Romania; claudiatoma2004@yahoo.com

**Keywords:** essential oils, plant extracts, phytochemicals, JAK/STAT Signaling Pathway, cytokines, anti-inflammatory activity

## Abstract

**Background/Objectives**: Essential Oils (EO), complex mixtures of organic compounds, exhibit a wide range of properties useful in the pharmaceutical, cosmetic, perfumery and agri-food fields. In particular, well-recognized EO anti-inflammatory properties push towards the investigation of the mechanisms underlying them. One of the signaling pathways targeted by EOs is the Janus Kinase Signal of Transducer and Activator of Transcription (JAK/STAT), whose hyperactivation is associated with inflammation, immune diseases and tumor progression. **Methods**: A comprehensive search on the major bibliographic databases was conducted; current findings and recent insights about the role of EOs in modulating the JAK/STAT Signaling Pathway were collected. **Results**: EOs derived from different plant species showed efficacy in attenuating the release of pro-inflammatory cytokines and mediators and in inhibiting phosphorylation of both JAK and STAT proteins. These results could be due to the EO’s multi-component nature and to the synergistic interaction occurring within these complex mixtures, both reflecting multi-target effects and modulations. Limitations concerning formulations, lack of standardization, efficacy and safety profiles, sustainable and eco-friendly approaches, and the gap between the literature and translation to the clinic were addressed. **Conclusions**: EOs represent emergent, promising and high-value candidates able to modulate the JAK/STAT Signaling Pathway.

## 1. Introduction

### 1.1. Essential Oils: An Overview

EOs, also known as “volatile oils”, “essence”, “etheric oils”, and “aetheroleum”, are defined as complex mixtures containing hundreds of organic compounds with low molecular weight and volatile characteristics at room temperature. Their chemical features are responsible for insolubility in water and solubility in organic solvents, including acetone and alcohol. EOs generally appear as colorless/pale yellow substances, liquid at room temperature, and are usually less dense than water [1,2]. EOs can be isolated from different parts of plants, such as leaves, bark, flowers, buds, seeds and peels [3]. In EO’s mixture of hydrocarbon compounds, bioactive aromatic substances, including terpenes (monoterpenes and sesquiterpenes), terpenoids (phenol alcohols, aldehydes, and ketones), benzene derivatives and miscellaneous others, can be recognized [4]. The specific composition strictly depends on two variables: internal factors (genetic, plant developmental stage and plant part used) and external factors (environmental conditions, post-harvest practices and extraction method) [5]. The methods used for EO extraction can be classified into classical/conventional and advanced/non-conventional extraction techniques. The first group includes steam distillation, hydrodistillation, hydrodiffusion and solvent extraction; the second one includes solvent-free microwave extraction, subcritical extraction liquid and supercritical fluid extraction. Although conventional methods are widespread, they require longer processing times and are characterized by lower extraction yield and efficiency. On the other hand, non-conventional methods produce higher extraction yields in less time and are also relatively low-cost [6]. EOs have always demonstrated a plethora of interesting pharmaceutical properties, including antiviral, antibacterial, antibiofilm, antifungal, antioxidant, anti-inflammatory, analgesic, anesthetic and anticancer properties. Furthermore, they are also seen as alternative biocontrol agents in the agricultural field, as green food preservatives, and as exploitable natural sources in both the cosmetics and perfumery industries [7,8,9]. Generally, their biological activity can be attributed to EO’s main components, although the minor ones can also exert antagonistic or synergistic activities, both in additive or potentiating terms [1,9,10,11]. The use of EOs is widely reported in traditional medicine, ceremonies, rituals, beauty and perfumery, as well as in food preservation. Sources have revealed that the first uses date back to 3000–2500 B.C. and are attributable to the Egyptian civilization. Further uses took place in China and India, while the first attempts at extraction came from the Arabs in the Middle Ages [12,13]. Currently, the global trade of EOs is experiencing renewed attention, driven by the growing demand for green, sustainable and natural products. The latest report, referring to 2023, indicated an EO global market size equal to USD 23.74 billion, with an expected annual growth rate of 7.6% in the period 2024–2030. The top four EOs are orange, eucalyptus, mint and lemon [14]. Concerning legislative control, EO regulation follows both national and international body rules, depending on the intended specific use. However, in order to address the issue of safety, the need for more stringent legislation is more urgent than ever [15,16]. In addition to the already well-established biological activities exerted by EOs, a new, emerging and promising body of evidence suggests that different EOs can modulate signaling pathways involved in a broad spectrum of pathologies, especially inflammatory diseases [17,18]. Inflammation, in fact, is modulated by fundamental molecular pathways such as Mitogen-Activated Protein Kinase (MAPK), Nuclear Factor-Kappa B (NF-kB), arachidonic acid signaling, and JAK/STAT [19]. Furthermore, EOs can be considered boosters for the immune system since they are able to modulate it by using antibodies, cytokines and dendritic cells and by stimulating immune-component cells (e.g., polymorphonuclear leukocytes, macrophages, dendritic cells, natural killer cells, and lymphocytes B and T) [11].

### 1.2. The Role of the JAK/STAT Signaling Pathway

**Figure 1:**
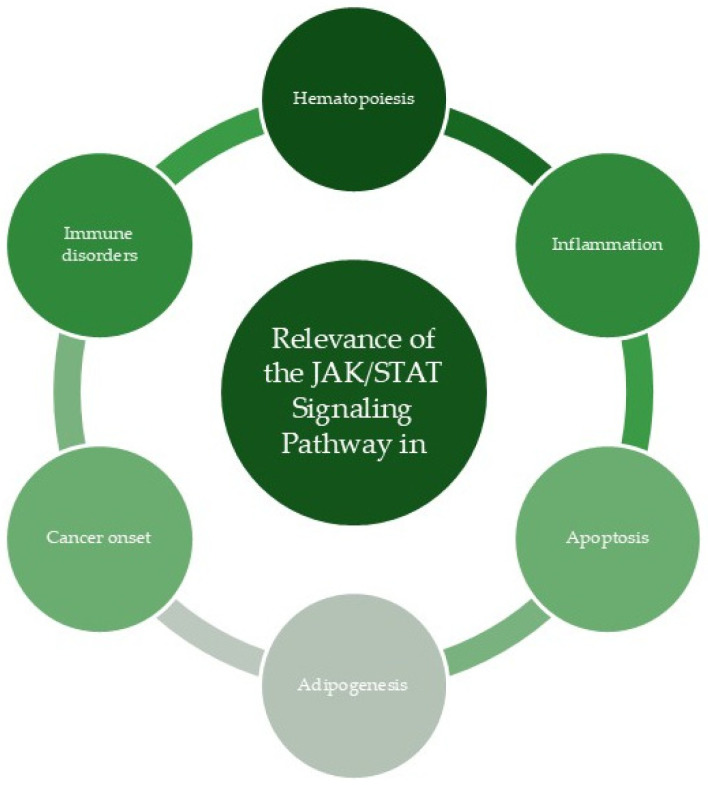
The JAK/STAT Signaling Pathway’s clinical relevance.

At the time of its discovery, the JAK/STAT cascade was considered an intracellular pathway that mainly modulates cytokines in mammals. Over time, evidence highlighted that this pathway also plays a key role in both the regulation of membrane proteins (e.g., integrin, G-protein-associated receptors, etc.) and downstream signaling [20]. The JAK family, in mammals, is composed of four members (JAK1, JAK2, JAK3 and tyrosine kinase 2 (TYK2)), while the STAT family includes a total of seven members (STAT1, STAT2, STAT3, STAT4, STAT5a, STAT5b, and STAT6) [21]. When cytokines bind the extracellular portion of the transmembrane receptor, receptor oligomerization occurs, and JAK transactivation takes place. Activated JAKs phosphorylate receptor cytoplasmic domains, allowing for STAT dimerization and translocation into the nucleus. At this point, STATs can start gene transcription [22,23,24,25]. Currently, it is known that the proper functioning of the JAK/STAT Signaling Pathway is crucial in different biological processes, and it plays a central communication role in cells, as the triggered downstream events are associated with hematopoiesis, inflammation, apoptosis, adipogenesis, cancer onset and autoimmune disorders (Figure 1) [26]. In fact, the processes mediated by the pathway are: immune regulation, cell proliferation, survival and apoptosis, hematopoiesis of both myeloid and non-myeloid cells, modulation of embryonic development and stem cell maintenance. Furthermore, crosstalk with other pathways significantly increases its clinical relevance [27]. Hematopoietic stem cells (HSCs), responsible for the production of the terminally differentiated blood cells, are mainly regulated by extracellular signals from the microenvironment, bone marrow and, overall, intrinsic cell signals, among which the JAK/STAT Signaling Pathway stands out. STAT protein phosphorylation modulates the hematopoiesis process by controlling HSC production, proliferation and self-renewal [28]. Tissue repairs and chronic wound pathologies are also factors modulated by both JNK/AP-1 and the JAK/STAT Signaling Pathway [29]. The JAK/STAT Signaling Pathway also exerts a central role in the regulation of the immune system [30]. Pathway activation, mainly driven by JAK2 and STAT5, is often linked to the proliferation, differentiation and maintenance of mammary gland epithelial cells [31]. Additionally, recent findings recognize the crucial role of the cascade in metabolic processes, including glucose tolerance, insulin sensitivity, adiposity, and energy expenditure [32].

### 1.3. Mutations and Aberrant Activation of the JAK/STAT Signaling Pathway

Over the years, both canonical and noncanonical JAK/STAT Signaling Pathway activation have been reported [33]. JAK/STAT Signaling Pathway activation is triggered by the binding of cytokines, growth factors, hormones, interferons (IFNs) and interleukins (ILs) [34]. Deregulation of the cascade, mainly occurring through genetic mutations, polymorphisms or hyperactivation, is associated with autoimmune and inflammatory disorders, beyond liquid and solid tumors [35]. It is known that the aberrant activation of the evolutionarily conserved JAK/STAT Signaling Pathway is involved in intricate biological processes, including inflammatory processes, immune response, cell proliferation, differentiation signals and epigenetics [36,37,38,39]. In aged hematopoietic systems and hematopoietic cancer, JAK often appears to be mutated. In fact, the dysregulation of the JAK/STAT Signaling Pathway, due to JAK1 and JAK3 somatic mutations, can be assessed in cutaneous T-cell lymphoma, while the translocation of the JAK2 gene is considered one of the causes of hematological diseases. JAK1 mutations can drive chronic eosinophilic leukemia and overall inflammatory syndromes. JAK1-inactivating mutations, instead, are mainly associated with the onset of solid tumors. Hyperactivation of JAK2, and the consequent activation of STAT5, is mainly linked to myeloproliferative neoplasm, polycythemia vera, thrombocytosis and myelofibrosis [28]. Aberrant JAK/STAT Signaling Pathway activation is involved in the pathogenesis of autoimmune disorders, including rheumatoid arthritis and systemic lupus erythematosus. Mutations in JAK3 hematopoietic cells are associated with aggressive lymphoproliferative disorders, while STAT3 exhibits a key role in breast cancer. In hormone-receptor-positive (HR+) breast cancer, for instance, the activation of the IL-6/JAK2/STAT3 pathway can lead to endocrine therapy resistance, while in Triple-Negative Breast Cancer (TNBC) models, STAT3 activation reflects tumor aggressiveness, immune evasion and poor clinical outcomes [35]. Also, STAT mutations have been investigated: STAT1 and STAT2 loss of function is mainly linked to damaged IFN responses, leading to susceptibility to both viral and mycobacterial infections. STAT3 mutations can be associated with Job’s syndrome, causing elevated IgE levels, skin abscesses and lung infections. STAT4, normally fundamental for Th1 cell differentiation and IL-12 signaling, when subjected to mutations, can be associated with an increased predisposition to autoimmune disorders. STAT5 mutations are mainly considered drivers of leukemogenesis, while STAT6 can lead to allergic syndrome and decreased defense against helminth infections [40].

### 1.4. JAK Inhibitors and Clinical Applications

Janus Kinase inhibitors (JAKis) represent a class of relatively new drugs for the treatment of inflammatory and immune-mediated disorders associated with JAK/STAT Signaling Pathway dysregulation. The therapeutic indications of these drugs include rheumatoid arthritis, ulcerative colitis, atopic dermatitis, inflammatory bowel disease, alopecia areata and vitiligo. They are also used to treat hematologic diseases, including myeloproliferative neoplasm, polycythemia vera and overall blood-associated malignancies. The first JAK inhibitor appeared in 2011. In 2023, nine additional inhibitors were approved [41]. JAKis represent a class of small-molecule drugs (<500 kDa) with rapid absorption and a positive pharmacokinetic profile. Thanks to their small molecular size, JAKis can pass cell membranes and act at a downstream level, blocking intracellular signal transduction modulated by cytokines. They can be administered orally or per transcutaneous and transmucosal methods [25,42]. Concerning efficacy profiles, different clinical trials have shown statistically significant results when compared to patients treated with placebos. The other side of the coin is represented by severe side effects: JAKis treatment is associated with vulnerability to infections (opportunistic infections and herpes zoster), perforation of the gastrointestinal tract, cardiovascular risk and an increase in venous thromboembolic diseases [43]. Furthermore, due to the limited number of available studies, the estimated risk of long-term adverse events is still unclear [44,45].

## 2. Results

### 2.1. Essential Oils Modulating the JAK/STAT Signaling Pathway in Inflammatory Response

#### 2.1.1. *Artemisia argyi* H.Lév. & Vaniot

*Artemisia argyi*, a widespread herbaceous plant species in Asia, is traditionally used in medicine for the treatment of hemorrhage, dysmenorrhea, bronchitis, arthritis and dermatitis (eczema). The beneficial properties mainly attributed to *A. argyi* include anti-inflammatory, immunomodulatory, antioxidant, analgesic, antimicrobial and anti-asthma activities. Chen and colleagues (2017) [46] investigated the anti-inflammatory potential of the plant in order to elucidate the underlying mechanism of action. *A. argyi* EO was obtained from leaves subjected to the hydrodistillation process, according to the *Chinese Pharmacopeia*. The phytochemical profile was investigated through Gas Chromatography–Mass Spectrometry (GC-MS): a total of 17 secondary metabolites were identified, with cineole, camphor, α-(-)-thujone and borneol the most abundant compounds. The EO’s effects on cell viability were investigated through a 3-(4,5-dimethylthiazol-2-yl)-2,5-diphenyltetrazolium bromide (MTT) assay. The EO, at concentrations of 10, 30, 90, and 270 µg/mL, did not affect cell viability in Lipopolysaccharide (LPS)-stimulated (1 µg/mL) RAW264.7 cells. *A. argyi* EO 270 µg/mL induced inhibition of nitric oxide (NO) and Reactive Oxygen Species (ROS) production by 50% and 86%, respectively. The best inhibitory activity against Prostaglandin E2 (PGE2) was exerted at the lowest concentration of 10 µg/mL. Furthermore, EO treatments significantly decreased levels of Tumor Necrosis Factor-α (TNF-α), IL-6, IL-10, IFN-β and Monocyte Chemoattractant Protein-1 (MCP-1) in a dose-dependent manner. Concerning an enzymatic screening assay, the EO, at all the tested concentrations, inhibited both inducible Nitric Oxide Synthase (iNOS) and Cyclooxygenase-2 (COX-2). No inhibition in p-MAPK, the NF-kB p65 subunit or the Activator Protein-1 (AP-1) subunit c-Jun was assessed at Western blot analyses, while dose-dependent suppression of p-STAT1, p-STAT3 and p-JAK2 in LPS-stimulated cells was observed without effects on the total JAK and STAT protein levels [46].

#### 2.1.2. *Magnoliae flos*

The flower buds of magnolia, commonly known as “Shin-i” in traditional Chinese medicine, are widely used for rhinitis, sinusitis and headaches. Although the biological properties of the Magnoliae ethanolic extract have already been demonstrated, the potential EO anti-inflammatory activity still needs to be better elucidated. In this regard, Wang and colleagues (2026) [47] investigated the potential of magnolia flower bud EO. This was phytochemically characterized with GC-MS, and a total of 35 compounds were identified, with eucalyptol (20.15%) the most abundant, followed by (-)-β-pinene (12.01%) and D-limonene (10.59%). Cell viability assessment was preliminarily performed through cell counting kit-8 (CCK-8) in RAW264.7 macrophages stimulated with LPS (1 µg/mL) in order to identify the sub-cytotoxic range. The data showed that magnolia flower bud EO treatment, in concentrations ranging from 50 to 300 µg/mL, did not affect cell viability. The EO treatment significantly and dose-dependently decreased NO release and ROS fluorescence intensity in RAW264.7 cells. Network pharmacology and Gene Ontology (GO) analyses predicted that magnolia flower bud EO would exhibit anti-inflammatory activity through its main bioactive compounds by modulating the JAK/STAT Signaling Pathway. These suggestions were confirmed by Western blot analysis, which revealed that magnolia flower bud EO suppressed p-JAK1 and p-STAT1 proteins by 68.63% and 47.61%, respectively [47].

#### 2.1.3. *Mentha x piperita* L.

The *Mentha* genus includes lots of aromatic species traditionally used both as food flavors and medicinal herbs. Kim and colleagues (2021) [48] investigated the effects of *Mentha piperita* EO, obtained from leaves through the hydrodistillation extraction process, on Particulate Matter (PM)-induced asthma. Asthma is an immune-related disease characterized by allergic sensitization, Th2 cell response to cytokine release, and the recruitment of eosinophils and neutrophils. Furthermore, the involvement of the IL-6-activated JAK/STAT Signaling Pathway was studied. Lung cancer A549 cells, previously sensitized with PM_10_, were treated with *M. piperita* EO in a concentration of 10^−3^/mL (*v*/*v* %). The data showed a significant mRNA reduction in TNF-α (81.9%), IL-1β (85.8%) and IL-8 (70.4%) cytokine expression. Similar data were observed for IL-5, IL-13, Matrix Metalloproteinase (MMP)-2 and MMP-9 suppression. *M. piperita* EO, at concentrations of 10^−5^, 10^−4^, and 10 ^−3^/mL (*v*/*v*%), reduced p-JAK2 by 34.0%, 37.7% and 76.4%, respectively, and p-STAT3 by 29.9% and 37.7% at the highest tested doses. No JAK1 modulation was observed. All these findings indicated that the inhalation or nebulization of peppermint EO could be effective in reducing asthma symptoms connected to air pollution exposure [48].

#### 2.1.4. *Chamaecyparis obtusa* (Siebold & Zucc.) Endl.

*Chamaecyparis obtusa* is a plant species native to Asia belonging to the Cupressaceae family, known for the ability to enhance psychological, cardiovascular and pulmonary functions. Leaves, properly pulverized, were subjected to hydrodistillation through a Clevenger apparatus. The obtained EO was characterized at GC-MS: α-terpinyl acetate (18.7%), sabinene (12.3%) and bornyl acetate (10.7%) were detected as the most abundant. The cell viability of both *C. obtusa* EO and sabinene was assessed through MTT assay in a RAW264.7 cell model previously treated with LPS. Neither compound, at concentrations lower than 200 µg/mL, affected cell viability. Dose-dependent NO inhibition, reduced c-Jun N-terminal kinase (JNK) and p38 phosphorylation were observed after treatment with both EO and sabinene. The EO significantly decreased the mRNA levels of IL-1β and IL-6 and reduced the phosphorylation of JAK1, STAT1 and STAT3, suggesting that it exerts its activity through MAPK and the JAK/STAT Signaling Pathway. Cytokine array analysis revealed that IL-1β, IL-6, IL-27, granulocyte–macrophage colony-stimulating factor (GM-CSF) and IL-1 receptor antagonist decreased after EO treatment [49].

#### 2.1.5. *Citrus medica* L. var. *sarcodactylis* Swingle

*Citrus medica*, commonly known as bergamot, is a precious edible medicinal plant. Its EO, derived from the peel, is widely recognized for its anti-inflammatory, antiallergic, analgesic and anticancer properties. Feng and colleagues (2023) [50] investigated the potential of bergamot EO in asthma and chronic respiratory diseases. Commercial bergamots were subjected to hydrodistillation; the phytochemical profile of the obtained EO was investigated through GC-MS; the major identified compounds were subjected to target prediction studies. The efficacy of *C. medica* EO was assessed in an ovalbumin-induced mouse model: the data showed that EO inhalation significantly reduced lung inflammation and IL-4, IL-5, IL-13 and IgE production. Furthermore, mRNA and protein expression data demonstrated that IL-6, IL-1β and TNF-α significantly decreased after in vivo treatment. *C. medica* EO cell viability was assessed by means of the CCK-8 test in mouse alveolar macrophages, demonstrating no toxicity up to 100 µg/mL. Among the predicted key targets, it seemed that the bergamot EO treatment could inhibit MAPK and the JAK/STAT Signaling Pathway [50].

#### 2.1.6. Cinnamon

Cinnamon EO is a traditionally used Chinese herbal medicine and a widely consumed edible plant. Its biological properties, including anti-inflammatory, antibacterial, antioxidant and antitumor effects, have already been widely demonstrated. Emergent insights concern the use of cinnamon EO to restore gut microbiota. This EO, traditionally obtained from plant branches and leaves, usually reveals (E)-cinnamaldehyde as a major compound. In one study, a commercial cinnamon EO sample was tested to counteract *Candida albicans* biofilm. The Minimum Inhibitory Concentration (MIC) was assessed at 260 µg/mL, while 80% of the biofilm reduction was identified at 530 µg/mL. Then, the cinnamon EO efficacy was tested in *C. albicans*-induced ulcerative colitis mice model through the administration of a strain suspension and a 3% Dextran Sulfate Sodium (DSS) solution. The model group treated with low, medium and high doses of cinnamon EO showed relief in terms of body weight and bloody stools compared to the control group. The colons of the control group showed a significant reduction in length, while cinnamon EO administration, especially at the highest tested dose, alleviated the symptoms. Furthermore, an increased variation in bacterial abundance, in terms of Bacteroidota and Verrucomicrobia within treated mice models, was observed. On the other hand, Firmicutes, Actinobacteriota, Cyanobacteria and *Escherichia coli* decreased. Then, the effect of the cinnamon EO on the JAK/STAT Signaling Pathway was investigated. As a result, IL-6, IL-17A, JAK2, STAT3 suppression was observed. Interestingly, JAK2 expression was reduced by 91% after the treatment with the lowest tested dose, while STAT3 showed remarkable inhibition (67%) at the highest cinnamon EO tested concentration. All these findings suggest that cinnamon EO is able to counteract *C. albicans* biofilm by inhibiting the JAK2/STAT3/IL-17A axis in mice [51].

### 2.2. Essential Oils Modulating the JAK/STAT Signaling Pathway in Inflammatory Skin Diseases

#### 2.2.1. *Rosmarinus officinalis* L.

*Rosmarinus officinalis*, commonly known as rosemary, is a perennial plant native to the Mediterranean area and traditionally used for allergies and overall inflammatory-related diseases. Li and colleagues (2023) [52] investigated the molecular mechanism underlying the EO use in atopic dermatitis. *R. officinalis* EO phytochemical profile was investigated through GC-MS analysis: (-)-camphor, eucalyptol and (+)-α-pinene appeared to be the most abundant compounds. SPF-healthy male KM mice, previously sensitized with an acetone olive oil solution containing 7% Dinitrochlorobenzene (DNCB), were randomly treated with 1% EO, 2% EO and 4% EO, diluted in acetone olive oil solution (ratio: 4:1). After treatment with *R. officinalis* EO, skin thickness, edema, keratosis, inflammatory cell infiltration and spinosus hypertrophy appeared to be significantly reduced. Serum analysis revealed that IL-6 and TNF-α levels were decreased. Moreover, IL-4, Cluster of Differentiation 4 (CD4) +T and phosphorylated JAK2 and STAT3 proteins appeared to be suppressed in animal skin tissues treated with *R. officinalis* EO. These findings suggested that *R. officinalis* EO showed efficacy in DNCB-induced atopic dermatitis in mice by regulating the JAK/STAT and the NF-kB pathways [52].

#### 2.2.2. *Thuja sutchuenensis* Franch.

*Thuja sutchunenesis* is a plant species belonging to the Cupressaceae family, widely used in traditional Chinese medicine for skin and liver disorders, rheumatism and bronchitis. Long and colleagues (2025) [53] investigated its EO effects on atopic dermatitis. *T. sutchuenensis* leaves were subjected to hydrodistillation in a Clevenger apparatus. The phytochemical profile of the obtained EO was investigated through GC-MS: 110 compounds were identified, with γ-terpinene and β-myrcene the most abundant. *T. sutchuenensis* EO was tested on C57 BL/6 mice. Animals were sensitized by injecting ovalbumin; then, the treated groups received a combination of ovalbumin and 5% and 10% EO. *T. sutchuenensis* EO application significantly decreased both lesion severity and scratching frequency. Furthermore, the abnormal proliferation and the differentiation of keratin in filaggrin appeared to be restored. The overall inflammatory response was modulated by 5% *T. sutchuenensis* EO: TNF-α, interferon (IFN)-γ and IL-1β production decreased, while the anti-inflammatory cytokine IL-10 increased. In addition, skin microbiota analysis was conducted: the data showed that animals treated with *T. sutchuenensis* EO preserved their overall microbial diversity. The beneficial activity of the product was also tested in vitro in RAW264.7 cells stimulated with LPS. Concentrations ranging from 0 to 100 µg/mL did not affect cell viability in the CCK-8 assay. *T. sutchuenensis* EO at 20 µg/mL inhibited NO, TNF-α, IL-1β, and IL-10 and downregulated COX-2, JAK1, p-JAK1, STAT3, and p-STAT3. All these findings indicated that *T. sutchuenensis* EO was able to modulate atopic dermatitis through the modulation of the JAK1/STAT3 Signaling Pathway [53].

### 2.3. Essential Oils Modulating the JAK/STAT Signaling Pathway in Cancer Models

#### 2.3.1. *Pinus mugo* Turra

Starting from the assumption that STAT3 inhibition counteracts cancer cell proliferation and viability signals, Thalappil and colleagues (2022) [54] investigated the potential of several different commercial EOs to downregulate STAT3 proteins. Among the 12 tested samples, which included *Pinus mugo*, *Lavandula angustifolia*, *Pinus sylvestris*, *Cupressus sempervirens*, *Hyssopus officinalis*, *Juniperus oxycedrus*, *Myrtus communis*, *Chamaemelun nobile*, *Melissa officinalis*, *Eucalyptus globulus*, *Pimpinella anisum*, and *Cananga odorata*, the first four mentioned EOs showed strong anti-STAT3 activities. Specifically, *Pinus mugo* EO, which significantly affected DU145 human prostate cancer cell viability with the lowest detected IC_50_ value of 70 µg/mL after 24 h of exposure, was carried forward for further analysis. The phytochemical profile of *P. mugo* EO was characterized using GC-MS, revealing a total of 22 compounds, among which β-caryophyllene (21.4%), bornyl acetate (13.5%), α-pinene (12.5%) and limone (10.9%) stood out. *P. mugo* EO treatment (50 µg/mL) decreased levels of Bcl-2, Myeloid Cell Leukemia-1 (MCL-1), Cyclin D1, X-linked inhibitor of apoptosis protein (XIAP), COX-2 and survivin, suggesting the involvement of apoptosis-related pathways. Additionally, it was observed that *P. mugo* EO inhibited DU145 migration cells in a dose-dependent manner in a wound healing assay. Moreover, interesting combination experiments also demonstrated a synergism between *P. mugo* EO and the well-known chemotherapeutic drug Cisplatin [54].

#### 2.3.2. *Chrysopogon zizanioides* (L.) Roberty

*Chrysopogon zizanioides*, known as “vetiver” or “khus”, is a perennial grass belonging to the Poaceae family, typically consumed as an infusion or decoction. Traditionally, vetiver roots have been used for fever, inflammatory disorders, dermatological conditions and gastrointestinal diseases. The EO from *C. zizanioides* roots represents a pillar in the fragrance and cosmetics industries but has also gained attention for the pharmacological properties, including antimicrobial, antioxidant, anti-inflammatory and anticancer activities, it exhibits. The EO was obtained from fresh roots through steam distillation, and its phytochemical profile was investigated through GC-MS. Forty compounds, largely sesquiterpenes and sesquiterpenoids, were identified overall. The EO was tested on human A549 lung adenocarcinoma, HCT-116 colorectal adenocarcinoma and human neonatal fibroblast (HDFn) cells. Cell viability tests showed a dose-dependent cytotoxicity in HCT-116 in the range 6.25–75 µg/mL. No cytotoxicity was assessed for HDFn cells, which demonstrated tolerance, even at high concentrations (300 µg/mL). On the other hand, an EO IC_50_ value equal to 167.82 ± 6.51 µg/mL was assessed in A549 cells. Then, the 10 most abundant compounds identified in *C. zizanioides* EO, Isovalencenol, α-Vetivol, Khusimol, Vetiselinenol, Germacrene D-4-ol, α-Vetivone, β-Vetivone, Rosifoliol, Isovalencenal, and α-epi-Muurolol were investigated to find hub targets. Regarding STAT3, molecular docking analysis revealed that Rosifoliol and α-Vetivone exhibited the best docking scores, with values of −5.19 kcal/mol and −5.09 kcal/mol, respectively. These findings aligned with the hypothesis that secondary metabolites from *C. zizainoides* EO could modulate oncogenic kinases and could act as a leading scaffold for STAT3 inhibitors [55].

#### 2.3.3. Navel Orange

Navel oranges are edible fruits belonging to the *Citrus* genus and the Rutaceae family. Yang and colleagues (2024) [56] investigated the potential anticancer properties of navel orange peel EO in TNBC models. Navel orange EO was obtained by matrix cold pressing, followed by molecular distillation aimed at removing waxes, carotenoids and pesticides, all substances that could negatively impact the sample’s biological activity. Navel orange EO concentrations, ranging from 6.25 µg/mL to 200 µg/mL, were tested with an MTT assay performed on MDA-MB-231 and MDA-MB-453 cells, respectively, demonstrating a dose-dependent cytotoxicity. CyclinB1 and CyclinD1 appeared to be significantly decreased in MDA-MB-231 cells after treatment with navel orange EO (100 µg/mL). Navel orange EO also induced apoptosis phenomena by modulating Bcl-2 and Bax-2 gene transcriptions and decreased levels of MMP2 and MMP9, both markers of tumor migration. In accordance with the Kyoto Encyclopedia of Genes and Genomes (KEGG) and GO pathways, the overall differentially expressed genes seemed to be related to the modulation of MAPK, JAK/STAT and FoxQ Signaling Pathways [56].

#### 2.3.4. *Thymus hirtus* subsp. *algeriensis*

*Thymus hirtus* subsp. *algeriensis* (Lamiaceae) is traditionally consumed as tea and widely used for its anti-inflammatory and anticancer properties. The EO was obtained by extracting aerial parts in a Clevenger apparatus. Guesmi and colleagues (2021) [57] investigated the phytochemical profile and biological properties of a wild *T. algeriensis* plant collected in Tunisia. GC-MS analysis revealed the presence of thymol, camphor, linalool, 2-carene, terpinene-4-ol, endo-borneol, eucalyptol, α-pinene and alloaromadendrene as the major compounds. The anticancer properties of *T. algeriensis* EO were evaluated both in vitro and in vivo. A cell viability assay (MTT) was performed at concentrations ranging from 0.5 to 50 pg/mL, exhibiting dose- and time-dependent cytotoxicity in HCT116 cells. *T. algeriensis* EO (0.5 pg/mL) induced moderate cytotoxicity, while the combination of EO and TRAIL, a new anticancer drug, increased cell death by 81%. TEO treatment induced the phosphorylation of extracellular-signal-regulated kinases (ERK) 1/2, c-jun, JNK, and p38 MAPK. Moreover, it inhibited both JAK and STAT3 phosphorylation, as well as cFLIP, Intercellular Adhesion Molecule-1 (ICAM-1) and Vascular Endothelial Growth Factor (VEGF) [57].

### 2.4. Essential Oils Modulating the JAK/STAT Signaling Pathway in Neurodegenerative Diseases

#### 2.4.1. *Dalberigia pinnata* (Lour.) Prain

*Dalberigia pinnata* (Lour.) Prain is a plant species typical of tropical and subtropical areas, used in Chinese traditional medicine for the treatment of wounds, burns and injuries. Qin and colleagues (2024) [58] investigated the effects of *D. pinnata* EO on Alzheimer’s Disease (AD) progression, both in vitro and in vivo. The EO was obtained through supercritical carbon dioxide extraction. Electroencephalogram signal analysis, conducted on 50 male and female healthy volunteers aged between 20 and 30, revealed that the EO inhalation induced δ and θ wave enhancement in both the frontal and parietal lobes, a result that can be identified as a state of deep relaxation. β wave enhancement, significantly observed in males, can be associated with better cognitive abilities and an increased β/α ratio in the temporal lobe region, suggesting an induced aroused and emotional state. Further studies were also conducted in vitro, in SH-SY5Y human neuroblastoma cell models. The data showed that the EO 0.04% *(v*/*v)* treatment did not affect cell viability and induced morphological changes in terms of breadth, brightness and boundaries. EO stimulation, followed by cell exposure to Aβ_1-42_ 10 µM, increased cell counts and induced an overall amelioration of cell morphology when compared to Aβ_1-42_ stimulus only. Treated groups showed reduced P-Tau, Glycogen Synthase Kinase (GSK), Aβ_1-42_, COX-2, IL-1β, TNF-α, IL-6 expression levels and increased Superoxide Dismutase (SOD) enzymes. Network pharmacology analysis revealed that *D. pinnata* EO could exert its anti-inflammatory potential by modulating the JAK/STAT Signaling Pathway. In fact, four strong binding complexes, including elemicin-TYK2, methyl eugenol-PARP1, methyl eugenol-JAK2, and methyl eugenol-TYK2, were identified [58].

#### 2.4.2. Cobaiba

Copaiba EO, deriving from the Copaiba tree oleoresin, is a product widely used in the food, cosmetics and wellness industries. The Food and Drug Administration (FDA) approved its use as a flavoring agent in foods and beverages. Urasaki and colleagues (2020) [59] investigated the Copaiba EO effect on the neuronal signaling pathway in SH-SY5Y human neuroblastoma cells. Copaiba EO was recovered through steam distillation starting from oleoresins of *Copaifera reticulata*, *Copaifera officinalis*, *Copaifera coriacea* and *Copaifera langsdorffii*. GC-MS analysis indicated α-cubebene, α-copaene, β-elemene, β-caryophyllene, γ-elemene, α-bergamotene, α-humulene, trans-cadina-1(6),4-diene, germacrene D, and β-bisabolene as the major terpenes. Treatment with Copaiba EO induced both time- and dose-dependent upregulation of the pI3K/Akt/mTOR Signaling Pathway; positive regulation was also observed for MAPK and the JAK/STAT Signaling Pathway. Surprisingly, and against the trend, all these findings indicated that Copaiba EO upregulated signals related to cell proliferation and metabolism, even though its effects could be reversed by treatment with the Cannabinoid 2 (CB2) receptor agonist [59]. In another study, conducted by the same research group, it was demonstrated that the EO was also able to upregulate MAPK and the JAK/STAT Signaling Pathway in HMC3 microglial cells and in Jurkat T-lymphocytes [60].

### 2.5. Essential Oils Modulating the JAK/STAT Signaling Pathway in Cardiovascular Diseases

#### *Lavandula angustifolia* Mill.

*Lavandula angustifolia* accounts for a long tradition of use due to the relevant biological properties it exerts. In a study conducted by Askarian-Amiri and colleagues (2022) [61], lavender EO was produced from flowers and investigated for its protective effects in an Oxygen–Glucose Deprivation (OGD)-induced injury in in vitro models and in the H9c2 cardiomyocytes cell line. Lavender EO (100 µg/mL) enhanced cell viability after OGD induction. Immunocytochemical analyses revealed that the EO treatment, at both 10 and 100 µg/mL, significantly decreased the expression levels of JAK2, STAT3 and Extracellular Signal-Regulated Kinase (ERK) proteins. Moreover, the lavender EO, post-OGD treatment, decreased apoptosis in cardiomyocytes [61].

The discussed EOs are summarized in Table 1.

## 3. Discussion

**Figure 2:**
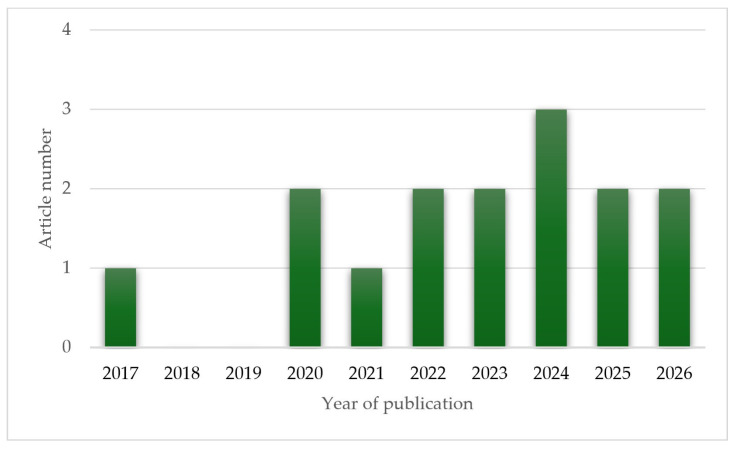
Conducted studies over the last few years.

**Figure 3:**
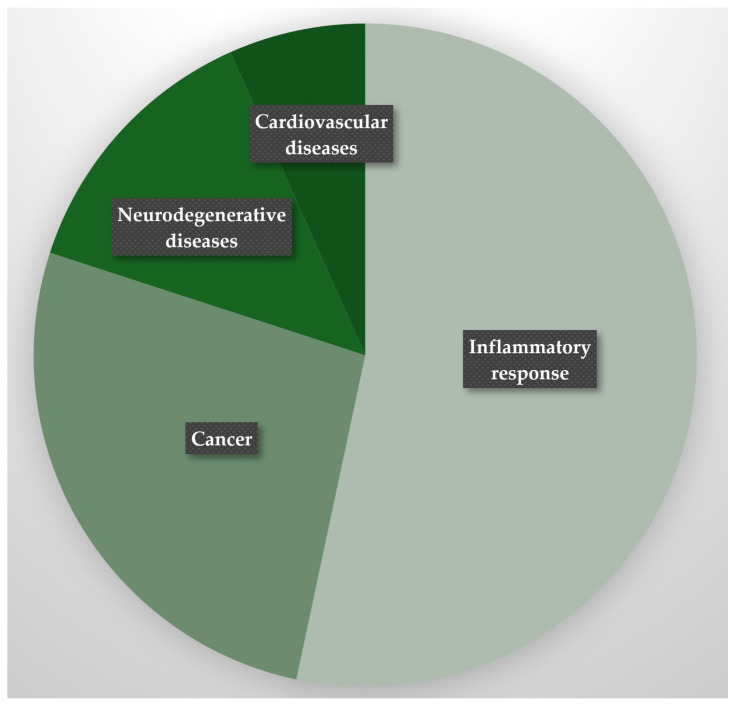
Potential application area distribution of EOs inhibiting the JAK/STAT Signaling Pathway.

The aim of this review was to collect and discuss current findings and recent insights about the emergent role of EOs in the modulation of the JAK/STAT Signaling Pathway (Figure 2). At first glance, the effects achieved by the evaluated studies fall into four areas of interest, mainly regulating inflammatory responses, cancer processes, neurodegenerative diseases and cardiovascular conditions (Figure 3). A total of eight examined studies, dealing with *A. argyi*, *Magnoliae flos*, *M. piperita*, *C. obtuse*, *C. medica* var. *sarcodactylis*, Cinnamon, *R. officinalis* and *T. sutchuenensis* EOs, refers to anti-inflammatory activity. Specifically, *M. piperita* and *C. medica* var. *sarcodactylis* showed remarkable potential effects against asthma, while most of the other mentioned works contributed to the understanding of EO’s anti-inflammatory potential in the LPS-stimulated RAW264.7 cell model. *R. officinalis* and *T. sutchuenensis* EOs deserve a specific mention, as they exhibit potential against atopic dermatitis, a recalcitrant inflammatory skin disease affecting a significant portion of the world’s population. The second largest portion of the evaluated studies regards EO’s anticancer potential via the downregulation of the JAK/STAT Signaling Pathway. *P. mugo* and *T. hirtus* subsp. *algeriensis* EOs showed efficacy in human prostate and colon cancer cell models, respectively, while a navel orange sample demonstrated efficacy in MDA-MB-231 and MDA-MB-453, two TNBC models. A very interesting effect was exerted by *C. zizanioides,* exhibiting efficacy against human A549 lung adenocarcinoma, HCT-116 colorectal adenocarcinoma and HDFn cell models. Regarding neurodegenerative diseases, *D. pinnata* and Copaiba EOs demonstrated powerful activity against neuroblastoma cell lines. A result that deserves particular attention, being inconsistent and in contrast to the trend of all the other analyzed samples, concerns Copaiba: its EO, in fact, is associated with an upregulation of the JAK/STAT cascade, which is responsible for signals of metabolism and proliferation. Lastly, among all its well-recognized biological properties, *L. angustifolia* EO, in the context of cardiovascular diseases, also showed promising effects in the modulation of the JAK/STAT Signaling Pathway.

The rationale behind the well-recognized positive effects exhibited by EOs can be found in the fact that plant-derived products are the result of a complex interplay between secondary metabolites, mainly including flavonoids, terpenes and alkaloids. Moreover, EOs are characterized by both structural diversity and multi-component activity, two features responsible for the elevated number of physiological targets, therapeutic indications and application areas of these precious mixtures [13]. In fact, due to the simultaneous presence of many different bioactive compounds, EOs are able to both exhibit effects on multiple molecular targets and establish synergistic potential when used in combination with other natural compounds [62,63]. Currently, around 3000 OEs are known and, among these, about 300 are commercially relevant [64]. The range of recognized beneficial activities attributed to EOs is rapidly increasing and ranges from antimicrobial effects to antioxidant and anticancer effects. Precisely because of EO’s extraordinary potential, issues concerning sample management, safety profile and efficacy need to be promptly evaluated and addressed. EO’s extraordinary appeal is mainly due to the widespread belief that a natural product is automatically safe, and this can create bias, affecting research [65]. It is important to generate awareness about the potential harmful or toxic effects of some plant secondary metabolites: different monoterpenes, for example, are neurotoxic, genotoxic or teratogens [66]. EO’s high versatility, reflected in the pharmaceutical, food and agriculture fields, is overall limited by relatively disadvantageous physical and chemical properties, including high volatility, thermal decomposition, poor water solubility and stability concerns [67]. Current advances in micro- and nano-encapsulation through different systems and carrier material types could partially overcome EO’s limits due to formulations and, simultaneously, improve functionality [68]. Nanotechnological formulations not only enhance bioavailability but, thanks to the use of biodegradable carriers and sustainable synthesis, also align with green chemistry global trends [69,70]. The FDA and the Environmental Protection Agency (EPA) classify EOs as Generally Recognized as Safe (GRAS), low-hazardous substances, overall able to protect human, animal and environmental health. This classification allows for the inclusion of EOs in cosmetics, food and feed products but does not establish their use as therapeutic agents. In fact, the required therapeutic doses, as well as low concentrations, can lead to serious problems at the respiratory and reproductive levels and can induce organ toxicity. Despite the fact that, over the past few decades, EOs have gained more and more popularity, a risk assessment of their uses still needs to be conducted. EOs have been reported as endocrine-disrupting chemicals (EDCs), which are exogenous agents that interfere with the production, release, transport, metabolism, binding or elimination of hormones in the body [2]. A study conducted by Sharma and colleagues [71] highlighted that prolonged exposure to some EOs could lead to a steroid imbalance [71]. Furthermore, EOs that are not properly diluted can even cause severe adverse reactions: phototoxic reactions, oral toxicity, eye irritation and, allergic contact dermatitis, a type IV delayed hypersensitivity reaction. Contact through the eyes and nose can cause redness, lacrimation and respiratory irritation. Skin contact may produce itching and rashes, requiring patch tests. Accidental ingestion can lead to gastrointestinal disorders and depression of the central nervous system [72,73,74]. Prolonged exposure to EOs could cause hepatotoxicity. Additionally, many EOs are associated with potential maternal toxicity, teratogenicity and embryotoxicity [75]. Several studies have investigated the synergistic effects of EOs with conventional drugs, even though data are limited to in vitro experiments [76]. EOs and their components have been tested, over the years, for their inducing or inhibiting effects on Cytochrome P450 (CYP) enzymes, the main route for drug elimination. CYP enzyme modulation, which occurs when both drugs and EOs are administered simultaneously and for a long period, is inevitable. The most important clinical issues arise when the difference between the toxic and effective doses of drugs is small [77].

Furthermore, significant chemical variability, as well as the need for standardized testing protocols and a lack of in vivo data, significantly affects the application and produces a relevant and non-negligible gap in the literature.

All the works evaluated in this review, for example, refer to in vitro studies only, highlighting the total absence of clinical studies [78,79,80]. GC-MS profiling is not only essential to identifying an EO’s chemical composition and correlating it with its biological properties but also to assessing product quality and authenticity. An EO’s chemical composition can undergo variations due to its species, growing conditions, harvesting period, applied extraction technique, and so on. Chromatographic analysis is also important for revealing adulterations in the product and for quantifying potential harmful compounds (e.g., allergens) or substances prohibited in certain amounts according to *Pharmacopeia* monographs and International Standardization Organization (ISO) norms [81,82]. According to Ferraz and colleagues [83], in the last 20 years, only 2% of published material about EOs regards their environmental impact, an unacceptable percentage in the current context and an issue that generates a gap in the literature that needs to be promptly filled. Studies concerning their impact on invertebrates and aquatic and terrestrial ecosystems need to be conducted. EOs, as widely discussed, are substances of plant origin and, therefore, are often associated with green, eco-friendly and sustainable practices, but this is not entirely true. Although most of the used EOs are described as non-toxic or slightly toxic at high concentrations, some of them result in toxicity even at concentrations lower than those defined by international guidelines. L(E)C_50_ values of 0.0336 mg/L, 0.0005 mg/L, and 0.0053 mg/L have been assessed for microalgae, crustaceans and fish, respectively [83].

Besides all these considerations, the literature data reports that EO effects produce a remarkable impact at a molecular level on cytokines, immunoglobulins and regulatory pathways. Pro-inflammatory cytokines and mediators appear to be significantly reduced after EO treatments [84].

Furthermore, despite recent advances and new drugs targeting the JAK/STAT Signaling Pathway, there is still a large gap in what is effectively understood about its cascade biology. For instance, the lack of a completely elucidated JAK crystal structure and, in consequence, the lack of interaction data could reflect a potential obstacle to discovery. Concerning STATs, one of the major issues regards their lack of specificity, considering the high number of cytokines able to activate them [85,86]. Additionally, JAKs and STATs are both regulators and proteins under the regulation of the epigenetic landscape [26]. Different JAK inhibitors, considered reasonably safe, have already been approved [87]. However, there are still many open questions about the JAK/STAT target’s specificity and the possibility of translating bench research into clinical practice [88]. Concerning the management of side effects, this aspect could be attenuated, at least in part, through the use or integration of plant-based products.

## 4. Methods

To conduct this research, a comprehensive search of Google Scholar, PubMed, Scopus and Web of Science was carried out. The following keywords “Essential Oil”, “Essential Oils”, “Volatile oil”, “Volatile oils”, “JAK/STAT”, “JAK/STAT Signaling Pathway”, “JAK”, “STAT” were matched and Boolean operators (“+”, “AND”) were inserted to maximize the retrieval. Papers eligible for inclusion had to match the following criteria: be published in peer-reviewed journals and be written in English. Review articles, conference papers, proceeding papers, editorials and book chapters were excluded. The first screening was conducted by evaluating titles and abstracts of the collected material. Duplicate papers were identified and removed, and then, the full-text of the remaining papers was evaluated. At the end of the screening process, other out-of-scope publications related to phytochemicals or secondary metabolites only, as well as those not related to total EO samples, were removed.

## 5. Conclusions

Although EOs can be retrieved only from 10% of the plant kingdom, they are attracting attention from the scientific community for their extraordinary potential and versatility [89]. Despite being associated for a very long time with aromatherapy, relaxation and stress relief only, evidence-based research has validated the plethora of pharmacological properties, including antiallergic, anticancer, immunomodulatory and overall anti-inflammatory effects, that EOs could exert. This suggests that EOs can be used in a holistic wellness approach [17,90]. On the other hand, available data continue to show evident limitations, including a lack of clinical studies, small sample sizes and few large-scale or long-term randomized controlled trials. Furthermore, chemical complexity, lack of standardization, batch-to-batch variations, missing pharmacokinetic data in humans, evaluation of dosage and administration routes, long-term safety and toxicological profiles, and uncontrolled risk of interactions need to be better evaluated and addressed as relevant challenges in the future [75]. Regulatory concerns about EO doses and usage need to be established to reduce potential adverse health effects [16]. This review applies a magnifying glass to the effect of EOs on anti-inflammatory activity and, more specifically, on the pro-inflammatory cytokines, mediators and signaling pathways underlying it. In particular, the JAK/STAT Signaling Pathway, known to be implicated in many biological processes, represented the main focus of this work. The retrieved material highlights that EOs exhibit an emerging and promising role as JAK/STAT modulators that deserves further research [84].

## Figures and Tables

**Table 1:** EOs modulating the JAK/STAT Signaling Pathway: botanical source, plant part used, extraction technique and potential application area.

EOs Botanical Source	Collection Site	Plant Part Used	Extraction Technique	Inhibition of the JAK/STAT Signaling Pathway Potential Application Area	References
*Artemisia argyi* H.Lév. & Vaniot	- *	Leaves	Hydrodistillation	Inflammatory response	[46]
*Magnoliae flos*	-	Flower buds	-	Inflammatory response	[47]
*Mentha x piperita* L.	-	Leaves	Hydrodistillation	Inflammatory response	[48]
*Chamaecyparis obtuse* (Siebold & Zucc.) Endl.	Gwangyangeup, Gwangyang-si, Jeollanam-do, Korea	Leaves	Hydrodistillation	Inflammatory response	[49]
*Citrus medica* L. var. *sarcodactylis* Swingle	-	Peel	Hydrodistillation	Inflammatory response	[50]
Cinnamon	-	-	-	Inflammatory response	[51]
*Rosmarinus officinalis* L.	-	-	-	Inflammatory skin diseases	[52]
*Thuja sutchuenensis* Franch.	National Nature Reserve Chongqing, China	Leaves	Hydrodistillation	Inflammatory skin diseases	[53]
*Pinus mugo* Turra	-	-	-	Cancer	[54]
*Chrysopogon zizanioides* (L.) Roberty	Torba Farm, Al Khor, Qatar	Roots	Steam distillation	Cancer	[55]
Navel orange	-	Peel	Cold pressing	Cancer	[56]
*Thymus hirtus* subsp. *algeriensis*	Orbata, Gafsa, Tunisia	Aerial parts	Hydrodistillation	Cancer	[57]
*Dalberigia pinnata* (Lour.) Prain	-	-	Supercritical carbon dioxide	Neurodegenerative diseases	[58]
Cobaiba	-	Oleoresin	Steam distillation	Neurodegenerative diseases	[59,60]
*Lavandula angustifolia* Mill.	-	Flowers	-	Cardiovascular diseases	[61]

* Data not available.

## Data Availability

No new data were created or analyzed in this study. Data sharing is not applicable in this article.

## Abbreviations

The following abbreviations are used in this manuscript:

AD	Alzheimer’s Disease
AP-1	Activator Protein-1
CCK-8	Cell Counting Kit-8
CD4	Cluster of Differentiation 4
COX-2	Cyclooxygenase-2
CYP	Cytochrome P450
DNCB	Dinitrochlorobenzene
DSS	Dextran Sulfate Sodium
EDC	Endocrine Disrupting Chemical
EOs	Essential Oils
EPA	Environmental Protection Agency
ERK	Extracellular-Signal-Related Cytokine
FDA	Food and Drug Administration
GC-MS	Gas Chromatography–Mass Spectrometer
GM-CSF	Granulocyte Macrophage-Colony Stimulating Factor
GO	Gene Ontology
GRAS	Generally Recognized as Safe
GSK	Glycogen Synthase Kinase
HR+	Hormone Receptor Positive
HSCs	Hematopoietic Stem Cells
IFN	Interferon
ICAM-1	Intercellular Adhesion Molecule-1
IL	Interleukin
iNOS	Inducible Nitric Oxide Synthase
ISO	International Standardization Organization
JAKi	Janus Kinase inhibitors
JAK/STAT	Janus Kinases/Signal Transducer and Activator of Transcriptions
JNK	c-Jun N-terminal kinase
KEGG	Kyoto Encyclopedia of Genes and Genomes
LPS	Lipopolysaccharide
MAPK	Mitogen-Activated Protein Kinase
MCP-1	Monocyte Chemoattractant Protein-1
MIC	Minimum Inhibitory Concentration
MMP	Matrix Metalloproteinase
MTT	3-(4,5-dimethylthiazol-2-yl)-2,5-diphenyltetrazolium bromide)
NF-kB	Nuclear Factor-kB
NO	Nitric Oxide
OGD	Oxygen–*G*lucose *D*eprivation
PGE2	Prostaglandin E2
PM	Particulate Matter
ROS	Reactive Oxygen Species
SOD	Superoxide Dismutase
TNBC	Triple-Negative Breast Cancer
TNF-α	Tumor Necrosis Factor-α
TYK2	Tyrosine Kinase 2
VEGF	Vascular Endothelial Growth Factor
XIAP	X-linked inhibitor of apoptosis protein
